# 

**Published:** 1885-07-20

**Authors:** 


Particularly indicated in Cholera Infantum, Diarrhœa and Stomach Disorders

THE CHAS H. PHILLIPS CHEMICAL CO., 30 Platt St., N. Y.

VOL. XV. JULY 20, 1885. NO. 7.

### THE SOUTHERN MEDICAL RECORD:

### A MONTHLY JOURNAL OF PRACTICAL MEDICINE.

Terms: $2.00 per Annum, in Advance; 4 Copies $6.00; Single Copies 20 Cents.

LIBRARY INDEX S.G.O.

*"QUICQUID PRÆCIPIES ESTO BREVIS."*

Address R. C. WORD, M.D., Managing Editor, Atlanta, Ga.

Entered at the Post-Office at Atlanta, as second-class matter.

